# Antimicrobial Resistance in Periodontal Disease: Rethinking Adjunctive Therapy in the Metagenomics Era, the Pressing Need for Precision Treatment and Periodontal Resistome Mapping

**DOI:** 10.1111/jcmm.71365

**Published:** 2026-10-30

**Authors:** Meera Alghafri, Lindsey Edwards, David Moyes, Luigi Nibali

**Affiliations:** ^1^ Periodontology Unit, Centre of Host Microbiome Interactions, Faculty of Dentistry, Oral & Craniofacial Sciences King's College London London UK

**Keywords:** antibacterial, antimicrobials, drug resistance, periodontitis, resistance, resistance genes, resistome, susceptibility, systemic antibiotics, therapeutic adjuncts

## Abstract

Systemic antibiotics are effective when used as adjunctive therapy in the treatment of periodontitis; however, their use must be balanced against antimicrobial resistance, which is a major threat to global public health. This review provides an expert synthesis of the literature and a field‐level overview of antimicrobial resistance in periodontal therapy, highlighting current knowledge and emerging directions, while examining how adjunctive antibiotic protocols may contribute to resistance within the periodontal microbiome through emerging metagenomic insights. To provide this synthesis, the PubMed database was searched for English‐language publications using combinations of the terms periodontitis, systemic antibiotics, antimicrobials, resistance, resistome, therapeutic adjuncts, resistance genes, susceptibility, antibacterial and drug resistance, and the findings are presented as a narrative review. The evidence indicates that a substantial proportion of patients with periodontitis harbour subgingival microbiota that are non‐susceptible to at least one antibiotic used in periodontal therapy. Across the included studies, antimicrobial resistance was most frequently reported in *Prevotella* and *Porphyromonas* species, while some studies also identified virulence‐associated factors in *Staphylococcus aureus* , a non‐classical periodontal organism detected within the subgingival microbiota. Overall, current evidence indicates a high prevalence of antibiotic resistance genes in the subgingival microbiota of patients with periodontitis and highlights the ecological consequences of adjunctive antibiotic use. These findings reinforce the need to optimize antibiotic prescribing protocols within an antibiotic stewardship framework and support further metagenomic investigations to refine understanding of resistance patterns, gene‐transfer mechanisms and microbiome disruption, with the aim of advancing precision‐based periodontal care.

## 1 Introduction

Periodontitis is a chronic multifactorial inflammatory disease of the supporting tissues of teeth. It is characterized by microbial challenge instigating a destructive host response leading to loss of periodontal tissue support, manifested through clinical attachment loss (CAL), alveolar bone loss, formation of periodontal pockets, gingival bleeding, and if left untreated, can possibly lead to tooth loss [1, 2].

According to the Global Burden of Disease 2021 Study, more than 1 billion people were affected by severe periodontitis, making it the sixth most prevalent disease globally [3]. Mild to moderate periodontitis affects an estimated 45%–50% of the global adult population, with severe periodontitis affecting around 10%–15% of adults worldwide. In addition to being a major cause of tooth loss, periodontitis also contributes to systemic health issues, including diabetes and cardiovascular diseases. Age, tobacco use, poor oral hygiene, diabetes and socio‐economic status are all likely to increase the incidence of periodontitis [4]. This is against the backdrop of the growing global threat of antimicrobial resistance (AMR). In 2019, bacterial AMR was estimated to be directly attributable to 1.27 million deaths globally and associated with 4.95 million deaths [5]. More recent global projections estimate that, by 2050, approximately 1.91 million deaths annually could be directly attributable to bacterial AMR, with approximately 8.22 million deaths associated with AMR [6].

In the management of periodontitis, oral hygiene instructions and non‐surgical periodontal therapy (NSPT) with manual and/or ultrasonic debridement remain the standard of care approach [7]. The aim of the NSPT is to eradicate and mechanically disrupt the bacterial biofilm responsible for disease initiation and progression. However, mechanical instrumentation can be suboptimal due to the inability to access areas of deep pockets and surface irregularities, such as furcation areas [8]. Hence, several adjunctive therapies (e.g., laser, photodynamic therapy, antimicrobial, airflow, host modulation and probiotics) were tested and studied in order to improve the microbiological and clinical outcomes of NSPT. Among these therapies, systemic and local antibiotics have been shown to be effective when used as an adjunct to NSPT [9, 10, 11, 12, 13]. Contrary to local antimicrobials, systemic antimicrobials have the ability to reach periodontal pathogens that may invade the host's tissue [14]. Even though few studies have investigated the prevalence of bacterial resistance to antibiotics before and after periodontal treatment, the existing evidence shows that the majority of periodontal pathogens have antimicrobial susceptibility regardless of the initial antibiotic resistance of some species [15, 16, 17]. The aim of this paper is to provide a summary of the current literature focusing on AMR in periodontal therapy.

## 2 Methods

To provide a synthesis of the current literature, a literature search was performed in the PubMed database. The search was conducted for each of the relevant topics addressed in this review. The following terms and their variants were searched either individually or in combinations: periodontitis, systemic antibiotics, antimicrobials, resistance, resistome, therapeutic adjuncts, resistance genes, susceptibility, antibacterial and drug resistance. Data from the identified papers were analysed and presented within the text or tables as indicated. The findings were presented in the form of a narrative review. A meta‐analysis was not performed due to the heterogeneity of the included studies.

## 3 Results

### 3.1 Microbiological Basis for Periodontal Treatment

Culture‐based evaluation has demonstrated that microbes residing in dental plaque clearly differ between healthy and diseased individuals. In periodontitis patients, the subgingival microbiota is mainly enriched with the ‘red complex’ organisms, including *Porphyromonas gingivalis, Tannerella forsythia* and *Treponema denticola* [18]. These organisms, together with other dysbiosis‐associated taxa, can sufficiently modify their environment in a manner favourable for their survival and persistence, driving microbial dysbiosis, which is crucial for the switch from health to disease. *P. gingivalis* in particular has been described as a keystone pathogen capable of reshaping the host inflammatory milieu and tipping the ecological balance towards pathogenicity [19].

‘The Keystone‐Pathogen Hypothesis’ suggests that disease becomes destructive when the overall microbial challenge exceeds the host's ability to neutralize pathogens. The disease is attributed to low‐abundance microbial pathogens, which increase the abundance and alter the composition of the normal microbiota [20]. Recent advances in molecular biology suggest that polymicrobial synergy and dysbiosis in a susceptible host, together with co‐adhesion and physiological compatibility, will lead to a balanced interaction between host immunity and the microbiota metabolism. This will be followed by colonization mainly by keystone pathogens such as *P. gingivalis*, enhancing the community virulence by their communication with accessory pathogens such as *Streptococcus gordonii*, disrupting the immune surveillance. The dysbiotic community, in turn, overgrows and increases in numbers, ensuing tissue destruction and further exacerbating the disease process [21]. Studies in the literature have elegantly demonstrated that in order to achieve therapeutic success, it is essential to foster the overgrowth of host‐compatible bacteria and suppress periodontal pathogens. Therefore, targeting the presence or absence of a few pathogens may not indicate the shift in subgingival microbial composition.

Previously, treatment of periodontitis has mainly focused on deep periodontal pockets. The rationale for this thought was the overall consensus that loss of attachment can occur after NSPT in shallow sites [22]. In fact, several studies reported that only a small percentage of untreated periodontitis showed further disease progression over time [23, 24, 25]. In addition, studies have shown that periodontal pathogens are concentrated in deeper pockets, which are the major focus of periodontal therapy [26]. However, the concept of treating only diseased sites has been debated, as these sites, in the case of periodontal disease progression and breakdown, may further jeopardize the retention of the adjacent teeth due to attachment loss [25]. Additionally, it has been documented that periodontal sites that are kept free of inflammation did not undergo further loss of attachment [27, 28]. On the other hand, a less well‐known factor is that even sites with shallow pockets can indeed be highly colonized with several periodontal pathogens [29]. Additionally, the presence of periodontal pathogens in the oral cavity has been studied in the literature. It was observed that red complex organisms can be found in the aerobic environment of the mouth, such as the oral cavity, tongue and saliva [30, 31]. The presence of strictly anaerobic pathogens in highly oxygenated environments might be explained by the protective effect of the biofilm structure [32]. In summary, successful periodontal treatment would require a complete shift in subgingival microbial composition, moving from a microbial profile associated with disease to a profile compatible with health.

### 3.2 Rationale for the Use of Antibiotics

An increased interest in the use of adjunctive antibiotic therapy in periodontology began in the late 1970s after the realization that disease progression is associated with bacterial colonization. Over time, clinicians realized that not all patients respond in the same way to periodontal therapy, and certain sites continue to experience periodontal breakdown despite the gold standard of care provided. In such sites, studies have confirmed that the combination of antibiotic therapy in conjunction with conventional periodontal treatment yielded a favourable clinical response [33]. These sites of continuous breakdown can be explained by the fact that NSPT alone may not provide enough sustainable change in subgingival microbial composition [34].

Many of the early clinical studies used tetracycline as an adjunct to periodontal therapy by using Listgarten and co‐workers regimen of 1 g/day of tetracycline for 14 days. These studies reported no added clinically significant effect of the adjunctive therapy compared to scaling and root planing alone [35, 36, 37]. On the other hand, other studies reported that tetracycline‐class antibiotic derivatives were able to modify the subgingival microflora of periodontitis patients [38, 39]. A question was raised whether we treat our patients or rather periodontal microbes with adjunctive antibiotics in periodontal therapy. A study was conducted aiming to explore the short‐term effects of mechanical periodontal therapy with or without adjunctive amoxicillin and metronidazole on the subgingival microbiome using 16S rDNA microbial community analysis. It was observed that when adjunctive antibiotics were used, there was a noticeable and significant microbiome shift reducing amplicon ribosomal sequencing variance (aRSVs) belonging to genera containing disease‐associated species, e.g., *Porphyromonas, Tannerella, Treponema and Aggregatibacter*, and by noticeably increasing health‐associated species. This shift was not found in the mechanical therapy group, which reflects that mechanical therapy alone did not statistically affect any disease‐associated taxa [10]. Harks et al. [40] investigated the long‐term impact of adjunctive systemic antibiotics on periodontal disease progression through prospective, randomized, double‐blind, placebo‐controlled multicentre trial comprising patients suffering from moderate to severe periodontitis. Results showed that the prescription of empiric adjunctive systemic antibiotics showed a small absolute, although statistically significant, additional reduction in further attachment loss following a 27.5‐month observation period. Moreover, a systematic review of 28 randomized clinical trials assessing the use of systemically administered antimicrobials as adjuncts to NSPT for up to 6 months and 1‐year post‐treatment concluded that the use of systemic antimicrobials as an adjunct to NSPT resulted in statistically significant greater probing pocket depth (PPD) reduction, pocket closure, clinical attachment level gain and bleeding on probing (BOP) reduction. However, more adverse events were reported in test groups using systemic antimicrobials [41].

To summarize, the rationale behind adjunctive therapies is that, although NSPT leads to a reduction in periodontal pathogens, it does not always modify the microbial profile sufficiently for the new, beneficial bacterial community to be established in a more definitive manner [42]. The choice of antibiotic to prescribe is about achieving the greatest antimicrobial activity with minimal drug‐related side effects, and in view of the ongoing global increase in antibiotic resistance, it is essential to optimize antibiotic use in periodontal therapy. A balance between desired effects and unwanted side effects should always be optimized. Clinicians should consider the patient's overall risk of periodontal disease when deciding whether to prescribe adjunctive antibiotics.

Empirical antibiotic therapy is the initiation of antibiotic therapy based on clinical experience and judgement while targeted therapy is the precise targeting of specific pathogens within a patient's microbiota identified by microbiology laboratory results. Currently, the treatment of periodontal disease using adjunctive therapies is empirically based. In fact, there is no evidence supporting that there is a distinct form of periodontal disease that would not benefit from amoxicillin and metronidazole combination [43]. Additionally, given the dysbiosis and microbial diversity observed in the subgingival microbiome of periodontitis patients [44], it is unclear to what extent the prescription of amoxicillin and metronidazole combination over a single‐drug treatment impacts the development of antibiotic resistance [45]. Moreover, unless susceptibility is explicitly tested, microbiological testing does not identify patients who are less likely to respond to an antibiotic because of resistance, and there is no evidence that guided microbiological therapy outperforms empirical antibiotic therapy [46].

A degree of individualization in antibiotic prescribing has been adopted by some trials based on periodontitis stage and grade [47]. In a randomized longitudinal study of *A. actinomycetemcomitans* positive and negative patients, it was concluded that the adjunctive use of amoxicillin and metronidazole resulted in a significantly lower number of sites with BOP and persistent probing depths > 4 mm compared to placebo (*p* < 0.01). The observed clinical improvements were independent of baseline microbiological status and baseline demographic characteristics. The results highlight that the detection of a single subgingival pathogen may not influence the outcome or the decision to use antibiotics [48].

While empirical therapy is effective when used as an adjunct to periodontal therapy, targeted antimicrobial strategies that maximize the therapeutic efficacy while minimizing the inappropriate use of antimicrobials are critically needed given the high prevalence of periodontal diseases. Finally, there is a future need to combine advanced diagnostic strategies such as functional metagenomics, metabolomics, metatranscriptomics and pharmacogenomics analyses to enable the application of precise targeted therapy in periodontal disease [49].

### 3.3 Emerging Crisis of AMR

A large number of randomized clinical trials and systematic reviews have clearly established that adjunctive systemic antibiotics combined with mechanical debridement, offer clinical improvements when compared with NSPT alone [11, 50, 51, 52, 53]. Although antibiotics were found to be able to induce microbial shifts in the subgingival microbiome when used as an adjunct, the ability to achieve long‐term stability of microbial shifts following periodontal therapy with or without antibiotics is a crucial question. Moreover, these additional benefits obtained from antibiotics must always be balanced against their undesirable side effects/adverse events (e.g., compliance, taste disturbance, renal and liver toxicity, nausea, vomiting, diarrhoea, colitis and opportunistic yeast infection) [41, 51, 54]. It is also imperative to highlight that antibiotics should only be used judiciously because AMR has become a major threat to global public health [55]. Such resistance may limit medical professionals' options for treatment, have negative economic impacts, and increase mortality [56]. In periodontitis, several bacteria that are associated with periodontitis, including *Aggregatibacter actinomycetemcomitans*, *Porphyromonas gingivalis* and *Tannerella forsythia* , have demonstrated resistance to common antibiotics, such as amoxicillin, metronidazole, clindamycin, azithromycin and amoxicillin/clavulanic acid [50, 57, 58, 59, 60]. Dentists are responsible for approximately 10% of antibiotic prescriptions worldwide, contributing significantly to the development of AMR [61]. According to the European Federation of Periodontology S3 level clinical practice guidelines, the overall use of antibiotics for both public health and individual patients should be taken into account in benefit versus harm analysis. In fact, long‐term effects of systemic antibiotic regimens on the faecal microbiome have been observed, including an increase in genes linked to antibiotic resistance [7]. Additionally, it is crucial to optimize antibiotic protocols in the context of antibiotic stewardship, taking into account a minimal bactericidal concentration and a minimal duration [62].

### 3.4 Overview of Antibiotic Resistance

Antibiotic resistance is defined as the ability of bacteria to survive and proliferate despite exposure to antimicrobial agents that would ordinarily inhibit or kill susceptible strains. It arises when microorganisms acquire genetically encoded resistance genes that diminish or negate the action of an antibiotic [63]. The success or failure of the antibiotic mainly depends on the susceptibility or resistance of the targeted bacterial species. In contrast, antibiotic susceptibility denotes the inability of bacteria or fungi to grow in the presence of one or more antimicrobial agents and is typically measured as the minimum inhibitory concentration (MIC) of an antibiotic (or drug) that completely inhibits growth [64].

#### 3.4.1 Mechanisms of Antibiotic Resistance Acquisition

Antibiotic resistance may occur through both intrinsic and acquired mechanisms:

- Intrinsic mechanisms, resulting from inherent genetic features or chromosomal mutations of natural recombination and integration into genomes that confer reduced susceptibility.
- Acquired mechanisms arising through horizontal gene transfer, including:
  ○ Conjugation (transfer of genes carried on plasmids, which are also known as mobile genetic elements).
  ○ Transduction (bacteriophage‐mediated transfer of DNA).
  ○ Transformation (direct uptake of naked DNA from the environment).
  ○ Other horizontal gene transfer events, including recombination and gene rearrangements [65].

#### 3.4.2 Cellular and Molecular Basis of Resistance

Bacterial resistance may be expressed through a variety of biochemical strategies, including:

- Modification of antibiotic targets, reducing drug binding affinity.
- Reduced permeability, such as inactivation or modification of the porin channel, limiting antibiotic entry.
- Enzymatic inactivation or degradation of the drug (e.g., β‐lactamases).
- Efflux pumps actively export antibiotics from the bacterial cell [66].

#### 3.4.3 Mobile Genetic Elements and Commensal Reservoirs

The transfer of antibiotic resistance genes (ARGs) located on mobile genetic elements (MGEs), including plasmids, transposons and integrons, facilitates dissemination of MGEs within and between bacterial species, thereby conferring resistance to bacteria and increasing the number of resistant strains. This enhances the emergence of resistant strains and establishes human commensal communities as significant reservoirs of ARGs [67, 68]. Importantly, oral bacteria can translocate to the gut and other body sites via the bloodstream, broadening the potential ecological impact of oral resistomes [69].

The overuse or inappropriate use of antibiotics can contribute to selection pressure and spread of MGEs and ARGs, increasing the risk, for example, of developing methicillin‐resistant *Staphylococcus aureus* (MRSA), vancomycin‐resistant enterococci (VRE), and multidrug‐resistant organisms (MDROs) [70]. Therefore, given the significant global threat to public health, numerous studies and systematic reviews emphasize the need for regional surveillance and population‐specific analyses to understand resistance patterns and optimize local antimicrobial stewardship, alongside addressing the global issue in the era of increasing AMR [55].

#### 3.4.4 Selective Pressure and Pharmacokinetic Considerations

A seminal genetic epidemiology study elegantly demonstrated that increased antimicrobial consumption exerts selective pressure that accelerates the emergence of antibiotic resistance, and that resistance declines slowly following reductions in drug use. The time scale for the emergence of resistance under constant selective pressure is shorter than the decay time after cessation or decline in the volume of drug use. Moreover, significant reductions in resistance require equally significant reductions in drug consumption [71].

The selective pressure of antibiotics is an important factor influencing the emergence of resistance in bacterial populations. Even subinhibitory antibiotic concentrations can select for resistant phenotypes; for example, low doses of minocycline are correlated with the emergence of strains showing reduced susceptibility in oral bacteria [72]. Thus, it has become critical to make the choice of antibiotic to treat oral infections, taking into consideration the concentrations that various drugs may achieve. Pharmacokinetic factors further influence resistance selection:

- β‐lactam antibiotics have a lower concentration in saliva than in serum, yet susceptibility patterns are not significantly impacted by these low serum levels [73].
- Erythromycin exhibits similar concentrations in serum and saliva to β‐lactams [74].
- Azithromycin, on the other hand, achieves markedly higher concentrations in saliva compared to serum [75], potentially contributing to its clinical behaviour and selection pressures in the oral environment.

Taken together, these findings emphasize that antibiotic resistance is a multifactorial process driven by genetic exchange, selective pressure, pharmacokinetics and microbial ecology. Understanding these mechanisms is essential for optimizing antimicrobial stewardship in periodontal therapy and for mitigating the broader public health consequences associated with antibiotic resistance.

### 3.5 Methods for Identifying Antibiotic Resistance Within the Oral Cavity

A range of laboratory and molecular methods are currently available for testing antibiotic resistance in the oral cavity. The most traditional and widely used approach is the MIC assay, which determines the lowest concentration of an antimicrobial agent (expressed in μg/mL) that completely inhibits visible growth of the test strain of an organism. However, several limitations of MIC testing are well documented:

- Inhibition of visible growth does not mean the organisms were killed.
- Only cultivable organisms can be assessed, excluding many oral taxa.
- MIC values depend on the dilution series used and therefore represent ranges rather than absolute thresholds.
- In vitro MIC values may not accurately reflect in vivo therapeutic efficacy [76].

An alternative method is the polymerase chain reaction (PCR), which amplifies specific deoxyribonucleic acid (DNA) segments using the DNA polymerase I enzyme [77]. PCR is both sensitive and rapid, important attributes for antibiotic diagnostics; however, its diagnostic power is restricted because it can only detect pre‐selected resistance genes, limiting its ability to capture the full diversity of the oral resistome [78].

Shotgun metagenomics sequencing provides a far more comprehensive strategy, a powerful method to sequence all genetic material within a sample, allowing characterization of the whole diversity of ARGs, the so‐called resistome. Metagenomics can provide a comprehensive sampling of all genes, yielding functional and taxonomic insights into microbial communities. It can identify novel or unexpected resistance determinants and also illustrate the function of the oral resistome and horizontal gene transfer in microbial communities in the absence of selective pressure. Metagenomics, therefore, offers a high‐resolution picture of resistance within the oral microbiome. However, despite its power, shotgun metagenomics is not yet suitable for routine use in clinical dental practice. It remains too expensive, requires sophisticated bioinformatic pipelines, and depends on the quality and quantity of the extracted DNA, limiting its integration into everyday periodontal diagnostics.

## 4 AMR in the Periodontal Literature: A Synthesis of Current Evidence

Recent studies have expanded our understanding of AMR within the subgingival environment. A large study recently investigated the antimicrobial susceptibility profiles and virulence genes of *Staphylococcus* species isolated from the subgingival biofilm of patients with periodontal health and disease. A total of 251 strains from 198 individuals with periodontal health, gingivitis and periodontitis were isolated. The results showed low susceptibility to at least one antimicrobial among most of the strains (78.2%), and 12% were multidrug‐resistant, including *S. aureus* , *S. epidermidis* , *S. saprophyticus* , *S. sciuri* and *S. warneri* . Additionally, an overall prevalence of 45% of subgingival staphylococci was observed. Penicillin G (60%), amoxicillin (55%) and azithromycin (37%) were associated with increased resistance rates [79].

A German surveillance study of 20,645 clinical isolates from dental and oral‐maxillofacial clinics found that *Streptococcus* spp. (36%) alongside *Staphylococcus* spp. (12%) were the most frequently isolated microorganisms in both hospitals and dental practices. However, lower proportions of penicillin and aminopenicillin resistance in both *Streptococcus* spp. and *Staphylococcus* spp. were observed in dental practices compared to hospitals (8%) in hospitals versus (2%) in dental practices for *Streptococcus* spp., and (6.9%) in hospitals versus (2.1%) in dental practices for *Staphylococcus* spp. Moreover, there was a relatively high resistance against clindamycin in both hospital and dental practices (17%–19%). However, it is important to highlight that in this study, anaerobic bacteria were largely absent in the microbiological sampling procedures, underlying diagnoses were not collected, and about more than half of the species of the oral flora could not be grown in vitro. In summary, this study underlines the importance of continuous efforts in optimizing antibiotic stewardship in both hospital and dental settings [80].

### 4.1 Phenotypic Resistance After Adjunctive Systemic Antibiotics

In a classic clinical trial, Feres et al. [81], determined the percentage and identified antibiotic‐resistant species in subgingival plaque and saliva samples from chronic periodontitis patients treated by NSPT followed by orally administered amoxicillin or metronidazole. Patients were randomly assigned to receive either 500 mg of amoxicillin orally, 3 times daily for 14 days or 250 mg of metronidazole orally, 3 times daily for 14 days. Results showed that most antibiotic‐resistant species detected during and after drug administration were also detected before their administration, suggesting that the transient increase in the proportion of resistant isolates was due to an overgrowth of resistant species present in the subgingival biofilm before exposure to the antimicrobial agent. The percentage of antibiotic‐resistant isolates returned to baseline levels 90 days after antibiotic administration, supporting the transient nature of these shifts.

### 4.2 Metagenomic Insights Into the Oral Resistome

Anderson et al. [82] combined shotgun metagenomics with culture‐based phenotyping to investigate the resistome and phenotypic resistance of oral biofilm microbiota from orally healthy, caries‐active, and periodontally diseased individuals. Sixty‐four ARGs to 36 antibiotics showed high prevalence of phenotypic resistance, particularly to tetracycline, macrolide‐lincosamide‐streptogramin (MLS) and beta‐lactam antibiotics. Moreover, shotgun analysis and phenotypic testing yielded different results. Another study analysed and compared the resistome profiles of 788 oral cavities worldwide with paired stool metagenomes. Resistance to tetracyclines, MLS antibiotics and beta‐lactams was observed. The oral cavity was found to contain the highest number of AMR genes. Furthermore, co‐occurrence analysis revealed contrasting associations of AMR genes between saliva and stool samples [83]. Caselli et al. [84] reported the presence of over 60 ARGs with lower prevalence towards beta‐lactam antibiotics and the highest prevalence for MLS and tetracycline resistance. While incredibly informative, it again demonstrates heterogeneity across sampling locations and methods; more metagenomic studies are urgently required.

### 4.3 Systematic Reviews and Cross‐Sectional Findings

A recent systematic review and meta‐analysis estimated the AMR profile in microorganisms present in periodontitis. The most cited microorganisms in the included studies were *Prevotella* and *Porphyromonas* species, and the virulence factors were related to *Staphylococcus aureus*. Ciprofloxacin showed the lowest frequency of resistance (3.4%), while ampicillin was associated with the highest resistance (39.5%). However, it must be highlighted that it was not possible to determine if the resistance in the microorganisms assessed is permanent or transient during cultivation. The main limitation in this review was the culture methods utilized among the studies, as in vitro susceptibility assessment does not accurately reflect the clinical efficacy and not all microorganisms are cultivable under artificial conditions [85]. Suggesting the field would greatly benefit from the availability of standards and standard methods. Other studies reported multidrug resistance in periodontal diseases which is probably related to the heterogeneous aetiology of periodontitis [57, 86, 87]. Another study using in vitro models aimed to characterize the *Staphylococcus aureus* strains isolated from periodontal lesions of patients and to determine gene expression involved in cell adhesion. Resistance to methicillin, dicloxacillin, ampicillin, cefotaxime and penicillin was observed among all the strains, and multidrug resistance to 6 of 12 antibiotics was reported [88]. Moreover, a cross‐sectional study aimed to investigate the composition of the oral microbiota from healthy individuals and patients with periodontal diseases, as well as the prevalence of ARGs. The proportion of ARGs was higher in diseased individuals but did not reach statistical significance. This result might be explained by the predominance of Gram‐negative species in periodontal lesions, which generally have a high horizontal transfer rate and exchange DNA among strains. There were no significant differences in taxonomic enrichment between the two groups. However, diseased samples had lower taxonomic diversity than healthy samples [89].

AMR is also related to the high presence of adhesion factors that contribute to biofilm formation. The biofilm can act as a reservoir of resistance genes, reduce treatment effectiveness, and increase microbial persistence [88, 89]. The sophisticated microbial intercommunication (quorum‐sensing) and the genetic exchange may lead to the horizontal transfer of genes, which can increase AMR [90].

### 4.4 Species‐Specific Susceptibility Profiles

Ardila and Bedoya‐Garcia [50] assessed a group of generalized periodontitis patients to assess the susceptibility of *Aggregatibacter actinomycetemcomitans* , *Porphyromonas gingivalis* , and *Tannerella forsythia* to amoxicillin, metronidazole, azithromycin and moxifloxacin. From a total of 76 patients, results reflected complete susceptibility of *A. actinomycetemcomitans* , *P. gingivalis* , and *T. forsythia* to moxifloxacin. Of the *A. actinomycetemcomitans* isolates, the highest resistance was observed in the metronidazole group, followed by amoxicillin and azithromycin. The *P. gingivalis* and *T. forsythia* samples showed comparable rates of resistance to amoxicillin, azithromycin and metronidazole. The absence of genotyping, single‐species testing, and testing at a single time point are the major limitations of the mentioned study [50]. On the other hand, a large German retrospective surveillance study of 7804 German adults diagnosed with periodontitis aimed to investigate changes in prevalence and antibiotic susceptibility of selected subgingival periodontal pathogens over 8 years. 63% of patients characterized isolates not susceptible to at least one of the antibiotics tested. The study concluded that there is a high prevalence of periodontal pathogens in the subgingival biofilm and revealed an incremental increase in isolates resistant to various antibiotics other than amoxicillin and metronidazole [91].

### 4.5 Metagenomic Studies of Disease States and Treatment Response

Gager [92] reported that two‐thirds of periodontitis patients carried ARGs to at least one of the antibiotics used in periodontal therapy, including β‐lactams, lincosamides, macrolides, nitroimidazoles and tetracyclines. It is crucial to highlight that the presence of genes does not necessarily suggest active resistance [93]. Using the same laboratory methods and shotgun sequencing, another study aimed to report the distribution patterns of ARGs and metal resistance genes of the dental plaque microbiota in the healthy state, periodontitis patients before treatment, and the resolved state of periodontitis patients after treatment. Periodontitis and NSPT significantly altered the composition of ARGs and metal resistance gene profiles, resulting in an increase in the number of both genes in dental plaque. The primary types of metal resistance genes observed in dental plaque microbiota are multimetal, iron, chromium and copper resistance genes. Additionally, a high abundance of ARGs was found related to bacitracin, beta‐lactams, MLS, tetracycline and multidrug resistance pathways [94]. A summary of clinical studies is presented in Table 1, and systematic review summaries in Table 2.

**TABLE 1:** Summary of clinical studies in the literature.

Study title	Year	Authors	Study design	Study aim	Location	Number of participants evaluated	Diagnosis	Method of assessment	Antimicrobial tested	Follow up period	Outcomes
Penicillin resistance in the subgingival microbiota associated with adult periodontitis	1986	S A Kinder/PMID: 3486882	Cross‐sectional study	To identify penicillin‐resistant and β‐lactamase‐producing species in patients diagnosed with periodontitis, and to test the association of recent exposure to penicillin and the recovery of resistant species from the microbiota	San Antonio, Texas	42 patients	Adult periodontitis	Culture	Penicillin	Cross‐sectional	There was a positive correlation between recent antibiotic exposure and the prevalence of penicillin‐resistant pigmented *Bacteroides* and *Veillonella* species. Pigmented *Bacteroides, Streptococcus*, and *Capnocytophaga* were found to produce β‐lactamase. Of all classes of antibiotics tested, metronidazole and clindamycin were the only agents active against all the penicillin‐resistant pigmented Bacteroides strains
Prevalence and antimicrobial susceptibility of Enterobacteriaceae, Pseudomonadaceae and *Acinetobacter* in human periodontitis	1990	J Slots/PMID: 2080069	Cross‐sectional study	To test the occurrence of non‐oral Gram‐negative facultatively anaerobic rods in advanced adult periodontitis	Philadelphia	427 patients	Advanced adult periodontitis	Culture	18 antimicrobials	Cross‐sectional	There was a variable susceptibility to the 18 antimicrobial categories tested but it was observed that all study strains showed high in vitro susceptibility to ciprofloxacin. Additionally, systemic ciprofloxacin therapy (500 mg BiD for 10 days) was effective in disease resolution and improved clinical periodontal parameters in 3 patients diagnosed with ‘refractory’ periodontitis
Susceptibility and resistance of plaque bacteria to minocycline	1990	B C O'Connor/PMID: 2324922	In vitro investigation	In vitro investigations	United Kingdom	55 strains of subgingival plaque bacteria	Periodontitis	Culture	N/A	7 weeks	In short term (6 h) exposure of bacteria to minocycline (8 μg/mL), there was significant reduction of a few periodontopathogens but there was minimal effect on the survival of *Veillonello parvula* and *Fusobacterium nucleatum* . Moreover, 7 weeks exposure to sub‐lethal concentrations of minocycline did not increase the MICs of most species except *Actinobacillus actinomycetemcomitans* NCTC 10981 and *Campylobacter concisus* NCTC 11485
Comparative evaluation of orally active antibiotics against community‐acquired pathogens: results of eight European countries	1996	W Cullmann/PMID: 8751263	Comparative study	To inspect the susceptibility of community‐acquired pathogens to penicillin G, ampicillin, amoxycillin plus clavulanic acid, cefaclor, cefuroxime, cefetamet, doxycycline and erythromycin	Italy, Germany, Finland, Netherlands, Switzerland, Greece, Spain, Hungary	13,173 pathogens	Non‐specified (community‐acquired infection)	Culture	Penicillin G, ampicillin, amoxycillin plus clavulanic acid, cefaclor, cefuroxime, cefetamet, doxycycline and erythromycin	Comparative study	There were no differences in the percentage of resistant isolates between the countries. However, high prevalence of high‐level penicillin‐G‐resistant *S. pneumoniae* isolates observed in Hungary and Spain. Cefuroxime susceptibility was observed in some of the low‐level penicillin‐G‐resistant strains, whereas all other β‐lactams studied showed complete cross‐resistance. The most active agent against Gram‐negative pathogen was cefetamet, while penicillin compounds showed the greatest activity against Gram‐positive pathogens
Systemic doxycycline administration in the treatment of periodontal infections (II). Effect on antibiotic resistance of subgingival species	1999	M Feres/PMID: 10599905	Clinical trial	To investigate the proportion and prevalence of doxycycline resistant species in subgingival plaque samples taken during and after doxycycline administration	United States	20 patients	Adult periodontitis	Culture	4 μg/mL doxycycline	90 days	There was an increase in the percentage of resistance isolates during doxycycline administration which was slowly decreasing 2 weeks, and 90 days later. There were also nonsignificant changes in resistance isolates in both plaque and saliva samples of the control subjects at the same time points. Doxycycline resistant strains were also detected prior to the administration of doxycycline in both plaque and saliva samples and in the control group. Approximately half of the species present at periodontal sites showed susceptibility to doxycycline administration at the end of 14 days, regardless of the increased resistance observed
Antimicrobial resistance in the subgingival microflora in patients with adult periodontitis. A comparison between The Netherlands and Spain	2000	A J van Winkelhoff/PMID: 10703651	Comparative study	To test the association between antibiotic usage and the level of antimicrobial resistance of the subgingival microflora of untreated patients diagnosed with adult periodontitis in The Netherlands and Spain	Netherland, Spain	31 patients (Spain), 30 patients (Netherlands)	Adult periodontitis	Culture	Penicillin, amoxicillin, amoxicillin and clavulanate, metronidazole, erythromycin, azithromycin, clindamycin and tetracycline	Comparative study	There was a statistically significant differences for penicillin, amoxicillin, metronidazole, clindamycin and tetracycline in both groups. There were also higher levels of resistance bacteria in the aerobic subgingival microflora of Spanish patients accounted for erythromycin, azithromycin and tetracycline. In Spanish patients, tetracycline resistance varied from 10 to 100% of the strains in periodontal pathogens. On the other hand, Dutch population showed only low percentage of tetracycline resistance in two species
β‐lactamase producing bacteria in the subgingival microflora of adult patients with periodontitis. A comparison between Spain and The Netherlands	2000	D Herrera/PMID: 10914894	Comparative study	To study the prevalence of β‐lactamase producing microflora in periodontitis patients in Spain and The Netherlands	Netherland, Spain	31 patients (Spain), 30 patients (Netherlands)	Adult periodontitis	Culture	Amoxicillin, and amoxicillin/clavulanate.	Comparative study	In the Spanish group, there was a trend of higher association between the prevalence and β‐lactamase producing microflora and higher antibiotic consumption history
Subgingival occurrence and antimicrobial susceptibility of *enteric rods* and *pseudomonads* from Brazilian periodontitis patients	2001	F C Barbosa/PMID: 11555308	Cross‐sectional study	To evaluate the presence of *Enterobacteriaceae* and *Pseudomonas* species in subgingival plaque from Brazilian periodontitis patients and the in vitro susceptibility to ciprofloxacin, doxycycline and amoxicillin/clavulanic acid	Brazil	80 patients	Periodontitis	Culture	Amoxicillin/clavulanic acid, ciprofloxacin and doxycycline	Cross‐sectional	All isolates showed high susceptibility to ciprofloxacin. There was a variable susceptibility to doxycycline and amoxicillin/clavulanic acid among all the organisms, except for *Pseudomonas aeruginosa* . Additionally, there was variable susceptibility patterns to the other antimicrobial agents tested
Antibiotic resistance of subgingival species during and after antibiotic therapy	2002	M Feres/PMID: 12390569	Randomized prospective clinical trial	To identify antibiotic‐resistant species in subgingival plaque and saliva samples from chronic periodontitis patients treated by scaling and root planing followed by orally administered amoxicillin or metronidazole	Brazil	20 patients	Chronic periodontitis	Culture	2 μg/mL metronidazole or 2 μg/mL amoxicillin	3 months	It has been concluded that resistance species noticed during or after drug administration was also present before drug administration. This highlights that this resistance might have occurred due an overgrowth of resistant species presented intrinsically. The percentage of antibiotic‐resistant isolates returned to baseline levels 90 days after antibiotic administration
β‐lactamase producing bacteria in adult periodontitis	2005	A.J. van Winkelhoff/DOI: https://doi.org/10.1111/j.1600‐051X.1997.tb00226.x	Cross‐sectional study	To study the occurrence of β‐lactamase production in periodontal bacteria	The Netherlands	23 patients	Adult periodontitis	Culture	Amoxicillin and amoxicillin + clavulanic acid	Cross‐sectional	A high frequency of β‐lactamase activity in sub gingival bacteria in adult periodontitis was observed. Species of the *Prevotella genus* were associated with the majority of β‐lactamase positive strains. It was also hard to rule out the mechanism of resistance
Periodontal disease as reservoir for multi‐resistant and hydrolytic *enterobacterial* species	2007	M O Gonçalves/PMID: 17451514	Cross‐sectional study	To assess antimicrobial resistance in chronic periodontitis patients and to isolate enteric rods	Brazil	80 patients	Periodontitis	Culture	AMP, ampicillin (10 μg); AMC, amoxicillin–clavulanic acid (20/10 μg); CAZ, ceftazidime (30 μg); CRO, ceftriaxone (30 μg); ATM, aztreonam (30 μg).	Cross‐sectional	High resistant to ampicillin (93.75%) and to amoxicillin/clavulanic acid (81.25%) was detected in the majority of the isolates. One *E. cloacae* strain presented resistance to tetracycline, cephalosporins (ceftazidime and ceftriaxone), aztreonam and sulfamethoxazole/trimethoprim. *Klebsiella pneumoniae* and *C. freundii* strain were susceptible to amoxicillin/clavulanic acid, but resistant to ampicillin. Susceptibility to cephalosporins and to aztreonam was noticed among most of the strains (93.75%)
Antibiotic resistance of periodontal pathogens obtained from frequent antibiotic users	2009	Carlos Serrano/PMID: 19839485	Cross‐sectional study	To determine the antimicrobial susceptibility of *Fusobacterium nucleatum* , *Prevotella intermedia* , and *Prophyromona gingivalis*, isolated from chronic periodontitis patients	Colombia	25 patients	Generalized‐moderate or advanced‐ chronic periodontitis	Culture	Amoxicillin, tetracycline, doxycycline, azithromycin and metronidazole	Cross‐sectional	Resistant to metronidazole was detected among all strains. Five strains showed resistance to tetracycline and azithromycin, and resistance to doxycycline and amoxicillin was observed in 2 strains. The strains resistant to amoxicillin were positive for β‐lactamase production. Antimicrobial resistance against metronidazole, was a common in this group of Colombian patients
Antibiotic resistance of subgingival species in chronic periodontitis patients	2010	C M Ardila/PMID: 20546113	Comparative study	To test the in vitro susceptibility of different species (*Porphyromonas gingivalis, Fusobacterium nucleatum, black‐pigmented Prevotella* spp. and *Aggregatibacter actinomycetemcomitans* ) in periodontitis patients to metronidazole, amoxicillin, amoxicillin/clavulanic acid, clindamycin and moxifloxacin	Colombia	76 patients	Chronic periodontitis	Culture	Metronidazole, amoxicillin, amoxicillin/clavulanic acid, clindamycin and moxifloxacin	Comparative study	There was a variable susceptibility in all antibiotics tested among all isolates, but sensitivity to amoxicillin/clavulanic acid and to moxifloxacin was observed in all isolates. *P. gingivalis* isolates showed resistance to amoxicillin, clindamycin and metronidazole in 25.49%, 23.52% and 21.56%, respectively. *F. nucleatum* highest resistance of 36.11% was towards clindamycin, 16.66% amoxicillin‐resistant, and resistance to metronidazole was noticed in 25%. Whereas *Prevotella* spp. were resistant to amoxicillin, followed by metronidazole, and clindamycin (35.55%, 26.66% and 22.22%, respectively). The least effective antibiotics against *A. actinomycetemcomitans* was metronidazole (88.88%), followed by clindamycin (83.33%), and amoxicillin (77.77%)
Antimicrobial resistance of aerobes and facultative anaerobes isolated from the oral cavity	2010	Ellen Cristina Gaetti‐Jardim/PMID: 21308284	Comparative study	To study antimicrobial resistance of both aerobes and facultative anaerobes in complete dentures patients, gingivitis, periodontitis, and periodontally health patients	Brazil	250 patients	41 complete dentures, 89 gingivitis, 70 chronic periodontitis and 50 periodontally healthy	Culture	Amikacin, ampicillin, amoxicillin/clavulanic acid, cefoxitin, cephalothin, chloramphenicol, ciprofloxacin, doxycycline, gentamicin, imepenem, meropenem, nalidixic acid, rifampin, and tetracycline	Comparative study	Carbapenems (meropenem and imipenem) was the most active antimicrobial drugs associated with low resistance (1.6%–2.3%). Microbial resistance was high to ampicillin, amoxicillin/clavulanic acid, cefoxitin, cephalothin, amikacin, chloramphenicol and nalidixic acid. The resistance to β‐lactams was found to be due to the synthesis of hydrolytic enzymes, particularly in gram‐negative enteric rods, while it was not proven that enterococci produce these enzymes. Of the 28.3% tested isolates, the combination of amoxicillin/clavulanic acid was not effective
Characterization of antibiotic resistance determinants in oral biofilms	2011	Seon‐Mi Kim/PMID: 21887642	Comparative study	To study the prevalence of antibiotic resistance from oral biofilms	United States	40 patients	20 healthy patients, 20 periodontitis patients	Polymerase chain reaction technique	Tetracycline, erythromycin, aminoglycoside, and B‐lactam	Comparative study	The prevalence of *tet (Q)*, *tet (M), cfxA*, and *blaTEM* was higher than 50% in both the patient and the healthy subject groups, with a tendency towards higher values in the patient group. A quantitative PCR analysis showed a positive association between periodontal disease and the relative amount of resistance. An increase in resistance genes was observed. Of them, the highest increase was observed for *erm (F)* of 145‐fold, 17‐fold for *tet (Q)*, 13‐fold for *tet (M)*, and 3‐fold for *cfxA*. It can be concluded that the resistome is more abundant in periodontitis patients
High prevalence of β‐lactam and macrolide resistance genes in human oral *Capnocytophaga* species	2014	Elodie Ehrmann/PMID: 24013195	Cross‐sectional study	To evaluate macrolide‐lincosamide‐streptogramin resistance determinants in the *Capnocytophaga genus* and to study the prevalence of β‐lactam resistance genes in human oral *Capnocytophaga species*	France	42 patients	14 haematology patients (HPs), 11 periodontitis patients (PPs) and 17 healthy volunteers (HVs)	Polymerase chain reaction technique	Amoxicillin, amoxicillin + clavulanic acid, cefotaxime, ceftazidime, clindamycin and erythromycin	Cross‐sectional	Only in a subgroup of the *C. sputigena* species showed blaCSP‐1 gene. MLS resistant was observed in 29% of isolates. Among the MLS‐resistant isolates, the *erm (F)* and erm (C) gene was observed (93% and 7%, respectively)
Antibiotic resistance in human chronic periodontitis microbiota	2014	Thomas E Rams/PMID: 23688097	Cross‐sectional study	To assess the occurrence of in vitro antibiotic resistance among periodontal pathogens in chronic periodontitis patients	United States	400 patients	Chronic periodontitis	Culture	Amoxicillin at 8 mg/L, clindamycin at 4 mg/L, doxycycline at 4 mg/L, and metronidazole at 16 mg/L	Cross‐sectional	A least one of the tested antibiotics showed resistance in 74.2% of patients diagnosed with chronic periodontitis. In vitro testing of doxycycline, amoxicillin, metronidazole or clindamycin showed resistance of *Prevotella intermedia/nigrescens*, *Streptococcus constellatus* , and *Aggregatibacter actinomycetemcomitans* . Forty‐Five patients showed *S. constellatus* resistance to both amoxicillin and metronidazole, and 9 patients showed ciprofloxacin‐susceptible strains of Gram‐negative *enteric rods/pseudomonads* which was also resistance to both amocicilin and metronidazole
Antimicrobial resistance and prevalence of resistance genes of obligate anaerobes isolated from periodontal abscesses	2014	Yi Xie/PMID: 23659425	Cross‐sectional study	To evaluate the prevalence of resistance genes and to assess the antimicrobial resistance profiles of obligate anaerobic bacteria from periodontal abscess lesions	China	41 patients	Periodontal abscess	Polymerase chain reaction technique	Clindamycin, doxycycline, amoxicillin, imipenem, cefradine, cefixime, roxithromycin, and metronidazole	Cross‐sectional	Clindamycin and roxithromycin resistance were 31.7%. Amoxicillin‐resistance strains accounted for 25%, followed by 16.7% to cefixime, 13.3% to metronidazole, and 6.7% to doxycycline. The *erm (F)* gene was present in 60.7% of the strains, and *cfxA* gene in 53.3% of the amoxicillin‐resistant strains. Eight metronidazole‐resistant strains showed two nim genes identified as *nimB*
Antibiotic resistance in human peri‐implantitis microbiota	2014	Thomas E Rams/PMID: 23551701	Retrospective study	To determine antibiotic resistance of bacteria isolated from peri‐implantitis lesions	United States	120 patients	Periodontitis + periimplantitis	Culture	4 mg/L of doxycycline, 8 mg/L of amoxicillin, 16 mg/L of metronidazole or 4 mg/L of clindamycin	Between 2006 and 2012	Clindamycin, amoxicillin, doxycycline and metronidazole resistance was observed in *Prevotella intermedia* /nigrescens or *Streptococcus constellatus* (46.7%, 39.2%, 25% and 21.7%, respectively). Only a few subjects (6.7%) showed resistance to both amoxicillin and metronidazole. Overall, the majority of the patients exhibited resistance to one or more of the tested antibiotics
*Viridians Group Streptococci* in Patients Undergoing Tooth Extraction	2015	Shree Dhotre/DOI: https://doi.org/10.15197/ejgm.01406	Cross‐sectional study	To assess the prevalence and susceptibility pattern of *Viridians group streptococci* infection in patients undergoing tooth extraction	India	80 patients	Periodontal disease/healthy patients	Polymerase chain reaction technique	Ampicillin, azithromycin, penicillin G, cefotaxime, erythromycin, tetracycline levofloxacin, ofloxacin, vancomycin, quinupristin/dalfopristin, levofloxacin, linezolid, clindamycin, clarithromycin, ceftriaxone, and cefepime	Cross‐sectional	The highest resistance was related to Lincosamides, followed by macrolides, tetracyclines, cephems, fluoroquinolones, oxazolidinones, streptogramins and penicillin. However, vancomycin and linezolid were both susceptible in all the 260 VGS strains. Mitis and salivarius exhibited multiple antibiotic resistance
Periodontal pathogens and tetracycline resistance genes in subgingival biofilm of periodontally healthy and diseased Dominican adults	2016	James R Collins/PMID: 26121972	Cross‐sectional study	To identify prevalence and tetracycline resistance genes in periodontal conditions of Dominican patients	Dominican Republic	77 patients	Gingivitis, CP and AgP	Polymerase chain reaction technique	Tetracycline	Cross‐sectional	The *fimA* II was the most prevalent genotype in patients diagnosed with periodontitis. Seven tetracycline‐resistant genes were observed. Tetracycline resistance genes *tet (Q)*, *tet (32)* and *tet (W)* were frequently detected, *tet (32)* was greater in periodontitis patients comparing to healthy patients
Detection of *cfxA2*, *cfxA3*, and *cfxA6* genes in β‐lactamase producing oral anaerobes	2016	Buhle Binta/PMID: 27119762	Cross‐sectional study	To identify β‐lactamase‐producing oral anaerobic bacteria in chronic periodontitis patients and to detect the genes responsible to produce these enzymes	South Africa	48 patients	Severe to moderate forms of chronic periodontitis	Polymerase chain reaction technique	3 μg/mL of amoxicillin with and without 0.75 μg/mL of clavulanic acid	Cross‐sectional	Results showed that β‐lactam antibiotics in MIC test showed resistance genes of 53 to 59% of isolates. Almost half of β‐lactamase‐producing strains carried the *cfxA* gene, mainly *Prevotella*, *Porphyromonas*, and *Bacteroides*. None of the strained carried the *blaTEM* gene. *CfxA* gene was classified among the strains to *cfxA2*, *cfxA3*, and *cfxA6*
Prevalence of β‐lactam (*blaTEM*) and Metronidazole (nim) Resistance Genes in the Oral Cavity of Greek Subjects	2016	Georgios Koukos/PMID: 27099637	Comparative study	To identify the prevalence of *blaTEM* and nim genes	Greece	154 patients	50 periodontally healthy patients, 52 cases of gingivitis and 52 cases of chronic periodontitis	Polymerase chain reaction technique	β‐lactam, metronidazole	Comparative study	*BlaTEM* gene was identified in plaque and tongue samples isolated (37%, and 60%, respectively). Gene was greater in tongue samples than plaque samples of both periodontally healthy group and chronic periodontitis group. Moreover, it was unable to detect the nim gene in all the samples
Biofilm Formation by Drug Resistant *Enterococci* Isolates Obtained from Chronic Periodontitis Patients	2017	Sonia Bhonchal Bhardwaj/PMID: 28273964	Retrospective study	To identify the biofilm formation ability of *enterococci strains* in periodontitis and their antimicrobial susceptibility	India	100 patients	Chronic periodontitis	Polymerase chain reaction technique	Amoxicillin, ciprofloxacin, erythromycin, vancomycin, gentamycin, teicoplanin	Retrospective study (Oct–Jan/2015)	Among the antibiotics tested, resistance to vancomycin was the highest (13.3%), followed by teicoplanin (13.0%), gentamycin and ciprofloxacin (8.7%), erythromycin (6.5%) and the least resistance was to amoxicillin (4.3%). Biofilm formation was significantly associated with increase resistance in the tested isolates (*p* < 0.001)
*Aggregatibacter actinomycetemcomitans* serotype prevalence and antibiotic resistance in a UK population with periodontitis	2017	Chaido Akrivopoulou/PMID: 28668698	Cross‐sectional study	To determine *Aggregatibacter actinomycetemcomitans* serotype prevalence and resistance in periodontitis patients	United Kingdom	50 patients	Aggressive periodontitis	Polymerase chain reaction technique	Penicillin (1 U), amoxicillin (2 μg), amoxicillin/clavulanic acid (30 μg), metronidazole (5 μg), clindamycin (2 μg), tetracycline (10 μg), ciprofloxacin (5 μg) and ceftazidime (30 μg)	Cross‐sectional	All isolates were resistant to penicillin and metronidazole, 87.5% were resistant to clindamycin, 83.9% to amoxicillin and 76.8% to ceftazidime. Tetracycline, amoxicillin/clavulanic acid were associated with the lowest rates of resistance (8.9%, and 14.3%, respectively). All the tested 56 isolates were susceptible to ciprofloxacin
Antimicrobial susceptibility pattern of oral isolates of *Aggregatibacter actinomycetemcomitans*	2019	Kishore G Bhat/PMID: 31516229	Cross‐sectional study	To study clinical isolates on a different antibiotic commonly used for oral/systemic therapy	India	40 patients	Localized aggressive periodontitis	Polymerase chain reaction technique	Amoxicillin, amoxicillin/clavulanic acid (2:1) (amoxiclav), tetracycline, doxycycline, clindamycin, azithromycin, moxifloxacin, cefazolin, ceftriaxone, cefuroxime, cefotaxime and metronidazole	Cross‐sectional	Susceptibility to moxifloxacin, cefotaxime and ceftriaxone was observed in all the isolates. *A. actinomycetemcomitans* strands showed resistance to metronidazole but were susceptible to all the antibiotics tested. Moxifloxacin was associated with intermediate sensitivity in one strain only
Azithromycin and erythromycin susceptibility and macrolide resistance genes in *Prevotella* from patients with periodontal disease	2019	Alexandre Arredondo/PMID: 30667163	Cross‐sectional study	To identify oral *Prevotella* spp. isolated from chronic periodontitis patients, to determine their susceptibility to azithromycin and erythromycin, and to evaluate the presence of macrolide resistance genes	Spain	52 patients	Chronic periodontitis	Polymerase chain reaction technique	Azithromycin, erythromycin	Cross‐sectional	Half of the isolates had the *erm (F)* resistance gene, and 19% carried erm (B). The most prevalent bacterial species detected were *Prevotella intermedia* and *Prevotella nigrescens* . Minimum inhibitory concentrations ranges were 0.016/0.032 to > 256 μg/mL for both antibiotics. MIC50 values for azithromycin was 1.5 μg/mL, and for erythromycin was 1 μg/mL. MIC90 values were similar (> 256 μg/mL) for both antibiotics
Antimicrobial susceptibilities of *Aggregatibacter actinomycetemcomitans* and *Porphyromonas gingivalis* strains from periodontitis patients in Morocco	2019	M Mínguez/PMID: 29967975	Cross‐sectional study	To determine the susceptibilities of *A. actinomycetemcomitans* and *P. gingivalis* , to amoxicillin, amoxicillin plus clavulanate, metronidazole, and azithromycin, and to compare the microbiological profiles of patients characteriseing *A. actinomycetemcomitans*, *P. gingivalis* or both.	Morrocco	45 patients	Periodontitis	Culture (epsilometric method)	Amoxicillin, amoxicillin plus clavulanate, metronidazole, and azithromycin	Cross‐sectional	All *A. actinomycetemcomitans* isolates exhibited susceptibility to amoxicillin, amoxicillin plus clavulanate. Of the isolates, 28% were resistant to azithromycin and 61.7% resistant to metronidazole. *P. gingivalis* was susceptible to all of antibiotics tested
Defining the oral microbiome by whole‐genome sequencing and resistome analysis: the complexity of the healthy picture	2020	Elisabetta Caselli/PMID: 32423437	A Single‐Arm, Single‐Visit observational study	To use whole genome sequencing for a site‐specific map of the oral microbiome of young adult healthy subject, to determine the peculiar microbial population of each oral micro‐habitat to understand the whole oral microbiome, and to assess the resistome of the healthy microbiome of the oral cavity	Northen Italy	20 patients	Young healthy subjects	100 ng of total extracted DNA were analysed by whole genome sequencing, the resistome of HOM population was characterized by real time quantitative PCR (qPCR) microarray performed on total DNA extracted from oral rinse specimens.	Aminoglycosides, β‐lactams, erythromycin, fluoroquinolones, macrolide‐lincosamide‐streptogramin B, tetracyclines, and vancomycin.	Cross‐sectional	Among drugs tested, 84 resistance genes detected to erythromycin, aminoglycosides, vancomycin, tetracycline, fluoroquinolones, macrolide‐lincosamide‐streptogramin B and β‐lactams. The highest prevalence was to macrolide‐lincosamide‐streptogramin and tetracycline resistance, whereas the lowest prevalence was towards β‐lactam antibiotics
Comparative In Vitro Resistance of Human Periodontal Bacterial Pathogens to Tinidazole and Four Other Antibiotics	2020	Thomas E Rams/PMID: 32046045	Cross‐sectional study	To study the prevalence and resistance of selected red/orange complex bacterial species from severe periodontitis patients	America	88 patients	Severe periodontitis	Culture	Tinidazole (16 mg/L), metronidazole (16 mg/L), amoxicillin (8 mg/L), doxycycline (4 mg/L) or clindamycin (4 mg/L)	Cross‐sectional	There was a significantly lower prevalence of tinidazole‐resistant species, and higher prevalence of amoxicillin, doxycycline or clindamycin‐resistant species
Bacterial diversity and prevalence of antibiotic resistance genes in the oral microbiome	2020	Viviane de Sousa Moreira Almeida/PMID: 32991620	Cross‐sectional study	To compare the diverseness of oral microbiome and antimicrobial resistance in periodontal health and disease	Brazil	110 patients	Periodontal disease/healthy patients	Polymerase chain reaction technique	Tested resistance genes (aac (fluroquinolones), *blaTEM*, *mecA* (β‐lactamase), erm (ERY), pbp2b (PEN))	Cross‐sectional	Majority of the samples (72.7%) tested positive for at least one of genes screened. More than half of variants were erm (58.2%). Lower prevalence of variant *mecA* (2.7%), pbp2b and aac (6 ‘) (1.8%) detected. There was no resistance genes related to both carbapenems or metronidazole
Resistance to β‐lactams and distribution of β‐lactam resistance genes in subgingival microbiota from Spanish patients with periodontitis	2020	Alexandre Arredondo/PMID: 32495224	Cross‐sectional study	To study the distribution of β‐lactamase genes and the multidrug resistance profiles in subgingival bacteria of periodontitis	Spain	130 patients	Generalized periodontitis Stage III or IV	Polymerase chain reaction technique	Tetracycline, chloramphenicol, streptomycin, erythromycin, and kanamycin	Cross‐sectional	The most identified genera were *Prevotella and Streptococcus*. Multidrug‐resistant was detected in most of the isolates. The most detected genes were bla*CfxA*, and *blaTEM* (24.8%, and 12.9%, respectively)
Characterization and Antimicrobial Susceptibility of Pathogens Associated with Periodontal Abscess	2020	Muhammad Irshad/PMID: 33003527	Cross‐sectional study	To analyse the clinical and microbiological characteristics, and antimicrobial susceptibility of periodontal abscess	Pakistan	45 patients	Periodontal abscess	Polymerase chain reaction technique	Amoxicillin, azithromycin, tetracycline and metronidazole	Cross‐sectional	Six *A. actinomycetemcomitans* isolates were tested, among them, four showed resistances to metronidazole, two to amoxicillin, and 1 to tetracycline. 10 *P. gingivalis* isolates were tested, and only one displayed resistance to Metronidazole. Similarly, out of 10 isolates of *P. intermedia* */nigrescens*, non‐susceptibility to amoxicillin was observed in two. All bacterial isolates showed susceptibility to azithromycin. Irrespective of the antimicrobial agent used, *A. actinomycetemcomitans* isolates were associated with a higher frequency of antimicrobial resistance compared to *P. gingivalis* and *P. intermedia/nigrescens*
Abundance and diversity of resistomes differ between healthy human oral cavities and gut	2020	Victoria R Carr/PMID: 32019923	Prospective	To compare the oral and the gut resistomes	China, Fiji, the Philippines, Western Europe, and the USA	1174 publicly available metagenomic samples	Healthy subjects	All processed metagenomes were mapped against nucleotide_fasta_protein_homolog_model from the antimicrobial resistance database CARD	N/A	Buccal mucosa (*n* = 87: 32 with one, 36 with two, 18 with three and 1 with six timepoints); dorsum of tongue (*n* = 91: 22 with one, 43 with two, 24 with three and 2 with four timepoints); dental plaque (*n* = 90: 23 with one, 43 with two, 20 with three, 1 with four and 3 with six timepoints); stool (*n* = 70: 13 with one, 33 with two, 21 with three, 2 with four and 1 with six timepoints)	The antimicrobial resistance genes were higher in the oral cavity compared to the gut. However, a lower diversity of resistance genes was observed in the oral cavity. There was a contrast in co‐occurrence analysis of antibiotic resistance genes‐species between stool and saliva samples. It has been concluded that resistance can differ in different body sites
Antimicrobial resistance of *Aggregatibacter actinomycetemcomitans* , *Porphyromonas gingivalis* and *Tannerella forsythia* in periodontitis patients	2020	Carlos‐Martín Ardila/PMID: 32169683	Cross‐sectional study	To test the susceptibility of common bacteria found in periodontitis patients *Aggregatibacter actinomycetemcomitans, Porphyromonas gingivalis* and *Tannerella forsythia*	Colombia	76 patients	Generalized periodontitis	Polymerase chain reaction technique	Amoxicillin, metronidazole, azithromycin and moxifloxacin	Cross‐sectional	Resistance to metronidazole was the highest among *A. actinomycetemcomitans* isolates (89.2%). Resistance to amoxicillin was (70.3%), and to azithromycin (40.5%). *P. gingivalis* samples showed lower resistance to amoxicillin (24.6%), azithromycin (21.3%) and metronidazole (24.6%). Around 21% to 25% of *T. forsythia* isolates were resistant to azithromycin, amoxicillin and metronidazole
Antibiotic Resistance of Human Periodontal Pathogen *Parvimonas micra* Over 10 Years	2020	Thomas E Rams/PMID: 33080856	Prospective	To identify the 10‐year changes in the resistance of subgingival *P. micra* in periodontal disease therapy	United States	600 patients	Severe periodontitis	Culture	Doxycycline (4 mg/L), clindamycin (4 mg/L), amoxicillin (8 mg/L) or metronidazole (16 mg/L)	2006 and 2016	Comparing to 2006, there was an increase in *P. micra* resistance species in 2016 (*p* < 0.001). Doxycycline was associated with 37.7‐fold increase (11.3% vs. 0.3% patients), and 23.7‐fold increase in clindamycin resistance (47.3% vs. 2.0% patients). There were minimal changes in resistance to amoxicillin (2.3% vs. 1.0% patients) and metronidazole (0% vs. 0.3% patients). In fact, in vitro testing of *P. micra* isolates showed susceptibility to both amoxicillin and metronidazole
Tetracycline and multidrug resistance in the oral microbiota: differences between healthy subjects and patients with periodontitis in Spain	2020	Alexandre Arredondo/PMID: 33391624	Comparative study	to determine the prevalence a of 11 *tet* genes and the multidrug resistance profiles of the tetracycline‐resistant species in healthy and periodontitis patients	Spain	259 patients	129 periodontally healthy subjects and 130 patients with periodontitis	Polymerase chain reaction technique	1 μg/mL of erythromycin (ERY), 64 μg/mL of kanamycin (KAN), 8 μg/ml of chloramphenicol (CHL), 128 μg/mL of streptomycin (STR), 2 μg/mL of cefotaxime (CTX), and 8 μg/mL of amoxicillin (AMX)	Comparative study	There was a variation in the frequency of *tet (M), tet (W)*, *tet (O), tet (32)* and *tet (L)*. The most detected gene was *tet (M)*. There was a high prevalence of multidrug resistance, and the greatest resistance was towards kanamycin (83.64%). There was a significant difference in the prevalence of resistance isolates between the two groups tested
Prevalence and antibiotic susceptibility trends of periodontal pathogens in the subgingival microbiota of German periodontitis patients: A retrospective surveillance study	2021	Karin Jepsen/PMID: 33934384	Retrospective surveillance study	To study the 8‐year changes in prevalence and susceptibility of periodontal pathogens	Germany	7804 patients	Moderate to advanced periodontitis	Polymerase chain reaction technique	10 μg amoxicillin (AML), 30 μg amoxicillin/clavulanic acid (AMC 2:1), 20 μg ampicillin/sulbactam (SAM 1:1), 30 μg doxycycline (DO), 2 μg clindamycin (DA), 15 μg azithromycin (AZM), 5 μg ciprofloxacin (CIP) or 5 μg metronidazole (MTZ)	8 years	Resistance to at least one of the antibiotics was observed in more than half of the patients (63.5%). There was a trend in decreasing susceptibility The data further revealed a trend towards decreasing susceptibility between 2008 and 2015 (*p* < 0.05)
Dental Plaque Microbial Resistomes of Periodontal Health and Disease and Their Changes after Scaling and Root Planing Therapy	2021	Yutong Kang/PMID: 34287005	Prospective	To study the antibiotic resistance genes and metal resistance genes in healthy, periodontitis, and resolved state	China	112 patients	48 healthy‐state (HS), 40 periodontitis‐state (PS; before treatment), and 24 resolved‐state (RS; after SRP treatment)	Shotgun metagenomics sequencing	N/A	N/A	Periodontitis and periodontal therapy displayed a significant alteration in the composition of antimicrobial resistance genes and metal resistance genes profiles. High abundance of antibiotic resistance genes was observed towards bacitracin, beta‐lactam, macrolide‐lincosamide‐streptogramin, tetracycline, and multidrug resistance genes. On the other hand, metal resistance genes found were multimetal, iron, chromium, and copper
Screening and Assessment of Antimicrobial Susceptibility of Periodontopathic Bacteria in Peruvian Patients with Periodontitis: A Pilot Study	2021	Miguel Angel Aguilar‐Luis/PMID: 33679978	Cross‐sectional study	To determine the aetiology and resistance profile of bacteria identified in a group of Peruvian patients with periodontal disease	Peru	8 patients	Severe periodontitis	Culture	Penicillin 10 U (P), ciprofloxacin 5 μg (CIP), azithromycin 15 μg (AZM), clindamycin 2 μg (CD), tetracycline 30 μg (TE), dicloxacillin 1 μg (DCX), ampicillin 10 μg (AMP), trimethoprim‐sulfamethoxazole 25 μg (SXT), amoxicillin 10 μg (AML), amoxicillin + clavulanic acid 30 μg (20/10) (AUG), and metronidazole 5 μg (MTZ)	Cross‐sectional	Resistance to penicillin and tetracycline was observed in *Actinomyces naeslundii* . Dicloxacillin resistance was observed in *Eikenella corrodens* . Moreover, *Rothia dentocariosa* showed resistance to amoxicillin + clavulanic acid and metronidazole but displayed susceptibility to trimethoprim‐sulfamethoxazole
Pathogenic microbial profile and antibiotic resistance associated with periodontitis	2021	R Ansiliero/PMID: 33505093	Cross‐sectional study	To assess the microbial profile in different stages of periodontitis, and to analyse the susceptibility profile of pathogenic agents	Brazil	18 patients	*n* = 15 individuals with periodontal disease (periodontitis stages I or II or III and IV) and *n* = 3 individuals healthy	Polymerase chain reaction technique	Tetracycline 30 μg; Chloramphenicol 30 μg; Imipenem 10 μg; Amoxicillin 10 μg; Azithromycin 15 μg; Cephalothin 30 μg; Clindamycin 2 μg and Cefotaxime 30 μg	Cross‐sectional	Periodontitis stages III and IV showed high prevalence of *Citrobacter freundii* , *Bacillus*, *Raoutella*, *Klebsiella azaenae*, *Streptococcus pneumoniae* , and *Pseudomonas*. *Streptococcus pneumoniae* , *Raoutella* spp. and *Enterococcus avium* were associated with multidrug resistance. The diversity of the bacterial community and periodontitis stage showed a statistically significant relationship
Prevalence and antimicrobial susceptibility of Gram‐negative *bacilli* in subgingival biofilm associated with periodontal diseases	2021	Laís Christina Pontes Espíndola/PMID: 33955542	Cross‐sectional study	To study the prevalence and resistant of Gram‐negative *bacilli* in different periodontal conditions, and to assess β‐lactamase and carbapenemase producers	Brazil	362 patients	*n* = 83 healthy; *n* = 74 gingivitis, and *n* = 205 periodontitis	Polymerase chain reaction technique	Ampicillin (10 μg), amoxicillin‐clavulanate (20/10 μg), cefazolin (30 μg), cefoxitin (30 μg), cefuroxime (30 μg), ceftriaxone (30 μg), ceftazidime (30 μg), cefepime (30 μg), amikacin (30 μg), gentamicin (10 μg), trimethoprim‐sulfamethoxazole (1.25/23.75 μg), ciprofloxacin (5 μg), imipenem (10 μg), piperacillin‐tazobactam (100/10 μg), aztreonam (30 μg), tobramycin (10 μg), ciprofloxacin (5 μg), ampicillin‐sulbactam (100/10 μg), amikacin doxycycline (30 μg), minocycline (30 μg), and levofloxacin (5 μg)	Cross‐sectional	Thirty‐six percent of all subgingival biofilm samples contained Gram‐negative *bacilli* which was more prevalent in periodontitis patients than periodontal health and gingivitis groups (24.1%, and 22.9%, respectively). Multidrug resistance was observed in 4.6% of the isolates, and resistance to at least 1 antimicrobial was observed in 60% of the strains
Frequency and expression of genes involved in adhesion and biofilm formation in *Staphylococcus aureus* strains isolated from periodontal lesions	2021	Alina Uribe‐García/PMID: 31300301	Cross‐sectional study	To study the occurrence of *Staphylococcus aureus* in periodontitis patients, and to assess the expression of genes involved in cell adhesion	Mexico	268 patients	Moderate or advanced periodontitis or gingivitis	Polymerase chain reaction technique	dicloxacillin (DC, 1 μg), penicillin (PE, 10 U), ampicillin (AM, 10 μg), erythromycin (E, 15 μg), cephalothin (CF, 30 μg), pefloxacin (PEF, 5 μg), levofloxacin (LEV, 5 μg), cefuroxime (CXM, 30 μg), cefepime (FEP, 30 μg), gentamicin (GE, 10 μg), tetracycline (TE, 30 μg), and trimethoprim‐sulfamethoxazole (SXT, 25 μg)	Cross‐sectional	Multidrug resistance to 6–12 antibiotics was observed in all strains. Resistance to methicillin, ampicillin, dicloxacillin, cefotaxime, and penicillin was detected in all strains. Most strains (60.4%) in PFGE analysis showed a distinctive pulsed‐field type. Unique combinations of antibiotic‐resistant phenotypes and virulence genes were detected
Antimicrobial Resistance and the Spectrum of Pathogens in Dental and Oral‐Maxillofacial Infections in Hospitals and Dental Practices in Germany	2021	Annika Meinen/PMID: 34149666	Retrospective observational study	To determine and compare the microbiological profile of clinical isolates from dental and oral‐maxillofacial medicine between dental practices and hospitals	Germany	20,645 clinical isolates from dental and oral‐maxillofacial settings, 299 patients	N/A	Culture	Penicillin, aminopenicillins (amoxicillin, ampicillin), markers for methicillin resistance (oxacillin, flucloxacillin), second‐generation cephalosporins (cefuroxim), third‐generation cephalosporins (ceftriaxon, cefotaxim, ceftazidim), clindamycin, macrolides (erythromycin, clarithromycin), fluoroquinolones (moxifloxacin, levofloxacin), and carbapenems (imipenem, meropenem, ertapenem)	2012 to 2019	The most prevalent isolate microorganisms in dental practice were *Streptococcus* spp. (36%) and *Staphylococcus* spp. (12%). Lower resistance to penicillin and aminopenicillin detected in dental practice
Prevalence of methicillin and β‐lactamase resistant pathogens associated with oral and periodontal disease of children in Mymensingh, Bangladesh	2022	Sharmin Sultana/PMID: 36015011	Cross‐sectional study	To study the prevalence of oral and periodontal pathogenic bacteria and their antimicrobial resistance pattern	Bangladesh	131 children	Periodontal disease (pain, loose teeth, swelling and pus discharges from teeth and gums, caries, gingivitis, aphthous ulcers, and broken and sharpened teeth)	Culture	Clindamycin (CD, 2 μg); azithromycin (AZM, 15 μg); erythromycin (E, 15 μg); amoxicillin (AMX, 30 μg); cefradine (CH, 25 μg); cefuroxime (CXM, 30 μg); cefixime (CFM, 5 μg); moxifloxacin (MO, 5 μg); ciprofloxacin (CIP, 5 μg); tetracycline (TE, 30 μg); chloramphenicol (C, 30 μg); and metronidazole (MT, 5 μg)	Cross‐sectional	Multidrug‐resistant was observed in all tested isolates with higher prevalence among *S. aureus* , *Streptococcus* spp. and *L. fermentum* . Resistance to 5–8 antibiotics was frequent in high proportion of isolates. β‐lactamase resistance genes (*blaTEM* and *cfxA*), and methicillin resistance gene (*mecA*) were detected in *S. aureus* , *Streptococcus* spp., and *L. fermentum isolates*
Relationship between phenotypic and genotypic resistance of subgingival biofilm samples in patients with periodontitis	2022	Moritz Sparbrod/PMID: 36671269	Cross‐sectional study	to identify the association between the phenotypic and genotypic resistance against ampicillin‐sulbactam, clindamycin, doxycycline and metronidazole in periodontitis patients	Germany	19 patients	Periodontitis	shotgun metagenomics sequencing	ampicillin‐sulbactam, clindamycin, doxycycline and metronidazole	Cross‐sectional	The highest prevalence gene detected was *tet (32)*, followed by *tet (M)*, *tet (Q)* and *tet (O)*. Resistance to metronidazole was not detected, neither the presence of the resistance gene nimI. Moreover, all erm genes identified were phenotypic resistant
Antimicrobial resistance and virulence of subgingival staphylococci isolated from periodontal health and diseases.	2023	Ana Paula Vieira Colombo/PMID: 37463947	Cross‐sectional study	to assess the prevalence, antimicrobial susceptibility profile, and the virulence genes of *staphylococci* species in patients with periodontal health and disease	Hospital at Universidade Federal do Rio de Janeiro (UFRJ), Brazil	545 patients	142 individuals with periodontal health, 101 with gingivitis and 302 with periodontitis	Isolated strains were identified by mass spectrometry. Antimicrobial susceptibility was determined by disk diffusion. The *mecA* and virulence genes were surveyed by PCR.	Antimicrobials tested included penicillin G (10 U), amoxicillin (10 μg), amoxicillin‐clavulanic acid (20/10 μg), cefoxitin (30 μg), azithromycin (15 μg), ciprofloxacin (5 μg), moxifloxacin (5 μg), clindamycin (2 μg), doxycycline (30 μg), minocycline (30 μg), chloramphenicol (30 μg), cotrimoxazole (sulfamethoxazole‐trimethoprim 1.25/23.5 μg), gentamicin (10 μg), linezolid (30 μg) and rifampin (5 μg)	cross‐sectional	Most of the strains showed low susceptibility to at least one of the antimicrobials tested. 12 percent of them were multidrug resistant including *S. aureus* , *S. epidermidis* , *S. saprophyticus* , *S. sciuri* and *S. warneri* . High resistance rates were observed towards penicillin G, amoxicillin, and azithromycin (60%, 55%, 37%, respectively)
The oral microbiota is a reservoir for antimicrobial resistance: resistome and phenotypic resistance characteristics of oral biofilm in health, caries, and periodontitis	2023	A C Anderson/PMID: 37179329	Clinical trial	To determine the resistome and phenotypic resistance of oral biofilm microbiota from orally healthy, caries active, and periodontally diseased patients	Multicentre trial, Germany	179 patients, 997 isolates	Healthy, caries active, periodontally diseased individuals	Shotgun metagenomic sequencing combined with culture technique	erythromycin (15 μg/mL), clindamycin (2 μg/mL), penicillin G (6 μg/mL), ampicillin (2 μg/mL), vancomycin (5 μg/mL), tetracycline (30 μg/mL), gentamycin (10 μg/mL), meropenem (10 μg/mL), ciprofloxacin (5 μg/mL), cefuroxime (30 μg/mL), tigecycline (15 μg/mL), and moxifloxacin (5 μg/mL)	2017–2020	High prevalence of phenotypic resistance was observed with 64 antimicrobial resistance genes to 36 antibiotics. Higher resistance was related to tetracycline, macrolide‐lincosamide‐streptogramin, and beta‐lactam antibiotics. Higher prevalence of antimicrobial resistant genes detected in healthy and caries active compared to periodontally diseased individuals
Antibiotic resistance genes in the subgingival microbiome and implications for periodontitis therapy	2023	Yann Gager/PMID: 37254939	Cross‐sectional study	To analyse the diversity of antibiotic resistance genes in periodontitis patients	Germany	39 patients	Periodontitis	shotgun metagenomics sequencing	beta‐lactam, lincosamide, macrolide, nitroimidazole, and tetracycline	cross‐sectional	Nineteen antibiotic resistance genes from 6 antibiotic classes were observed. More than half of the patients showed antibiotic resistance genes to at least one of the antibiotic classes relevant to periodontal therapy (beta‐lactam, lincosamide, macrolide, nitroimidazole, and tetracycline). Majority of the patients were non‐susceptible to macrolide
Emergence of antibiotic‐resistant *Porphyromonas gingivalis* in United States periodontitis patients	2023	Thomas E Rams/PMID: 37998786	Prospective	To study changes over a 20‐year period in the antibiotic resistance patterns of subgingival *P. gingivalis* in periodontitis patients	United States	2193 patients	Severe periodontitis	Culture	4 mg/L for clindamycin and doxycycline, 8 mg/L for amoxicillin, and 16 mg/L for metronidazole	20 years/three time points: 1999–2000 (936 patients), 2009–2010 (685 patients), and 2019–2020 (572 patients)	There was 5‐fold increase in clindamycin‐resistant *P. gingivalis* between 2019 and 2020 as compared to 1999–2000. Twenty‐eight‐fold increase of *P. gingivalis* resistance to amoxicillin between 2019 and 2020 as compared to 1999–2000 was also observed. Moreover, antimicrobial resistance to metronidazole, metronidazole plus amoxicillin, and doxycycline remained low and stable over the 20‐year period

**TABLE 2:** Summary of systematic reviews in the literature.

Review title	Year	Authors	Objective	Main findings
Clinical significance of bacterial resistance to tetracyclines in the treatment of periodontal diseases	1995	G Greenstein/PMID: 8558393	To provide background information for the discussion concerning resistance to tetracyclines	Resistance to tetracycline was reported in 12% of the total cultivable subgingival microflora of periodontitis patients. It was also reported that following tetracycline therapy resistance might increase, and resistant organisms may be transient or persist for a long period of time
The acquisition of antibiotic resistance in the periodontal microflora	1996	Clayb Walker	To address antibiotics resistance and to provide information on how previously susceptible bacteria have become resistance to commonly used antibiotics	It was concluded based on the current data that transfer of tetracyclines and penicillin resistance genes across genus and species lines or animal hosts is possible. The past decade has shown an increase in the prevalence of oral microflora antibiotic resistance to tetracyclines and penicillin
Mechanisms of action of systemic antibiotics used in periodontal treatment and mechanisms of bacterial resistance to these drugs	2012	Geisla Mary Silva Soares	To analyse the mechanisms of action and resistance of the antibiotics used in the periodontal treatment	Resistant to penicillin in the subgingival microbiota is generally low. This resistance can be due to the alteration of the penicillin‐binding proteins, decreased permeability of the bacterial cell to the antibiotic, and the synthesis of β‐lactamases. Moreover, resistance to tetracyclines and macrolides can be due to restricting the access to the cell, synthesis of tetracycline/macrolide‐inactivating enzymes and modifying the ribosome to prevent effective binding of the drug. Hence, periodontal pathogens can become non‐susceptible to these drugs. Finally, acquired resistance to metronidazole has rarely been reported, as metabolic activation by strict anaerobe microorganisms is required
Distribution of genes related to antimicrobial resistance in different oral environments: A systematic review	2015	Ludmila Coutinho Moraes	To study antibiotics resistance genes distribution in different human oral sites	There is a high prevalence of antimicrobial resistance genes in saliva, dental biofilm, and endodontic infections. Resistance was associated with tetracycline and β‐lactam prescriptions. The most frequent prevalence genes were *tet (M)*, *tet (Q)*, *tet (W)*, and *cfxA*
Oral Gram‐negative anaerobic *bacilli* as a reservoir of β‐lactam resistance genes facilitating infections with multiresistant bacteria	2015	Clarisse Dupin	To report the main mechanisms of resistance to β‐lactam antibiotics and their prevalence in Gram‐negative oral bacilli and to address their gene transfer observed in the oral cavity	Antimicrobial treatment has been modified due to the detection of β‐lactamases in clinical samples. The role of mobile genetic elements on the expression of β‐lactam resistance genes is not fully understood. It can occur as a result of overexpression of efflux pumps or the synthesis of β‐lactamases
Metagenomic Insights into Transferable Antibiotic Resistance in Oral Bacteria	2016	S Sukumar	To study the role of metagenomics in the investigation of the oral microbiome, the transmission of resistance in the oral microbiome, the distribution and diversity of resistance genes, and the resistance profiles of specific pathogens	Oral commensal bacteria can act as a reservoir of antimicrobial resistance genes. A higher presence of antimicrobial resistance genes was detected in recent metagenomics studies. It has been showed that horizontal gene transfer is the main route for antibiotic resistance in the oral biofilm. Moreover, many antimicrobial resistance genes are located on mobile genetic elements which further aid horizontal gene transfer
A review of virulence factors, pathogenesis, and antibiotic resistance in *Staphylococcus aureus*	2016	Al‐Mebair	To describe *S. aureus* . virulence factors, pathogenesis, and antibiotic resistance	A form of β‐lactamases (penicillinase) is found to be produced by penicillin‐resistant strains which is responsible for the hydrolyzes of the b‐lactam ring of penicillin. Additionally, in large plasmid, β‐lactamase (blaZ) gene was located, which is a transposable element often found with additional antimicrobial resistance genes (e.g., gentamicin and erythromycin)
Metronidazole resistance and nim genes in anaerobes: A review	2019	Corentine Alauzet	To report metronidazole resistance rates among anaerobes in the past 20 years	Surveillance studies are needed to study nim gene‐mediated resistance. The location of nim genes on mobile genetic elements is highly important. Nim genes is also emergent in multidrug resistant anaerobes, which is also associated to resistances to other anti‐anaerobes (piperacillin‐tazobactam, clindamycin and carbapenems). This resistance is challenging when deciding the most effective drug of choice
Methicillin‐Resistant *Staphylococcus aureus* in the Oral Cavity: Implications for Antibiotic Prophylaxis and Surveillance	2020	Eric S Donkor	To study the antibiotic resistance in the oral flora with regard to methicillin‐resistant *Staphylococcus aureus*	There is a high prevalence of Methicillin‐resistant *Staphylococcus aureus* in the oral cavity, which is a public health concern as it has the potential for Methicillin‐resistant *Staphylococcus aureus* transmission. There is an essential need to consider re‐thinking before the administration of prophylactic antibiotics to at‐risk dental procedure candidates. New therapeutic alternatives are urgently needed in the context of antimicrobials resistance
The antimicrobial susceptibility of *Porphyromonas gingivalis* : Genetic repertoire, global phenotype and review of the literature	2021	Georg Conrads	To test the in vitro susceptibility of *P. gingivalis* strains in patients with advanced forms of periodontitis	There was no resistance observed in amoxicillin + clavulanate, cefoxitin and imipenem. Intermediate susceptibility/resistance to amoxicillin and metronidazole was reported among only few strains. Treatment failure may occur when macrolide, **c**lindamycin, fluoroquinolones, and tetracyclines are used in the treatment of *P. gingivalis‐*associated mixed anaerobic infections due to the presence of resistance genes erm(B), *erm(F)* and *tet(Q)*. In order to optimize antibiotic stewardship, it is essential to conduct periodical antibiotic susceptibility tests
Antimicrobial resistance of microorganisms presents in periodontal diseases: A systematic review and meta‐analysis	2022	Flávia Casale Abe	To review the antimicrobial resistance in microorganisms, present in periodontal diseases	Antimicrobial resistance in periodontal diseases was highly prevalent in *Prevotella* and *Porphyromonas* species. The highest virulence factors reported were associated to *Staphylococcus aureus* . The most frequent resistance reported was towards ampicillin (39.5%) and the lowest frequency to ciprofloxacin (3.4%). Macrolides genes were the most cited
Prevalence of antibiotic resistance genes in the oral cavity and mobile genetic elements that disseminate antimicrobial resistance: A systematic review	2022	Laura Brooks	To evaluate the prevalence of antibiotic resistance genes and to identify mobile genetic elements associated with the resistance	*tet(M)* prevalence was similar between healthy, periodontitis and peri‐implantitis patients. In addition, poor oral hygiene was associated with higher resistance genes. There was significant resistant to tetracycline and erythromycin in *Enterococcus faecalis* isolates. Moreover, studies showed that beta‐lactam resistance genes (*blaZ* and *cfxA*) were higher in periodontal pathogens and erythromycin resistance genes (*mef(A/E)*) were higher in normal oral flora
Antibiotic resistance in patients with peri‐implantitis: A systematic scoping review	2022	Carlos M Ardila	To observe antibiotic resistance in peri‐implantitis patients	In peri‐implantitis patients, resistance to tetracycline, metronidazole, and erythromycin was reported in *Porphyromonas gingivalis* and *Fusobacterium nucleatum* . Clindamycin and doxycycline resistance was found in *Aggregatibacter actinomycetemcomitans* . Resistance to other antibiotics varied in other bacterial isolates such as *Tannerella forsythia* , *Parvimonas micra* , and *Prevotella intermedia/nigrescens*. However, no resistance to the combination of amoxicillin and metronidazole was reported across the included studies
Antibiotic resistance in the microbiota of periodontitis patients: an update of current findings	2024	Ethan Ng	To investigate the current understanding of antibiotic resistance in the subgingival microbiota of periodontitis patients	*Porphyromonas gingivalis* , *Prevotella intermedia* , *Prevotella denticola* , *Prevotella melaninogenica* , *Fusobacterium nucleatum* , *Tanerella forsythia*, *Aggretibacter actinomycetemcomitans*, *Streptococcus constellatus* , *Streptococcus intermedius* , and *Parvimonas micra* displayed antibiotic resistant isolates, but did not exceed 10% to any specific antibiotics. *Aggretibacter actinomycetemcomitans* was reported with increase resistance towards amoxicillin. Amoxicillin, clindamycin, and metronidazole were associated with the highest prevalent of resistance. Geographical locations impacted the distribution of antimicrobial resistance

## 5 Future Consideration and Concluding Remarks

A major conceptual shift in the diagnosis and management of periodontal diseases is underway. Microorganisms inhabit and play unique roles in all ecosystems. Advances in microbiome science have highlighted the complexity and ecological organization of microbial communities across human body sites. Prosser et al. [95] highlighted the importance of ecological principles in understanding microbial communities, alongside subsequent advances in high‐throughput technologies for analysing microbial community structure and function. In periodontitis, pathogenesis involves a complex interplay between periodontopathogens and the host immune system. Dysbiotic and highly organized climax biofilms enable resistance to host elimination, during which periodontal pathogens can sufficiently modify their environment to create optimal conditions for growth, thereby favouring their survival. As a result, collateral damage to the periodontium occurs due to immune‐subversive and pro‐inflammatory cytokines produced by mature subgingival biofilms. The majority of this damage is thought to be host‐dependent, relative to genetic predisposition and environmental factors. There is an ongoing exploration of the individual's own genome for identifying metagenomic codes for health and disease [96]. The concepts of keystone pathogens, along with polymicrobial synergy and dysbiosis, are considered the basis for future therapeutic options in periodontal disease. Moreover, overuse of antibiotics has contributed to the emergence of resistant species. These features underscore the pressing need for precision‐based periodontal treatment strategies that minimize unnecessary antibiotic exposure and target specific microbial or functional signatures rather than applying population‐wide regimens.

Emerging approaches include specifically targeted antimicrobial peptides, e.g., C16G2, which was found capable of removal in vitro of cariogenic *Streptococcus mutans* , alongside reconstruction of the multispecies communities [97]. Another alternative approach is host‐modulation strategies inhibiting destructive inflammation of the host through blocking immune‐evasion pathways of pathogens or limiting inflammatory exudate‐derived nutrients. Vaccination against pathogens would be a highly effective alternative to antibiotics. Another possible method for treating periodontitis could address the underlying inflammatory imbalance by recruiting regulatory T‐cells. Sustained release of the chemokine CCL22 reduced the clinical scores of periodontitis as well as hard‐ and soft‐tissue destruction in mice and dogs [98]. An alternate strategy to antibiotic treatment is also the manipulation of the microbiome through oral or faecal microbiota transplant, probiotics or synthetic biology by genetic modification of bacteria. These methods aim not only to suppress pathogenic taxa, but also to rebalance host–microbial interactions and restore ecological stability.

However, significant scientific and methodological challenges must be addressed before precision periodontal therapy becomes routine. A notable limitation of the current evidence in the literature is the substantial methodological heterogeneity across studies investigating AMR in periodontal pathogens. Evidence is derived largely from closed‐ended culture‐based techniques, which detect only a fraction of the oral microbial community and underestimate resistome complexity. Moreover, differences in sampling procedures, including variations in subgingival plaque collection, storage conditions and patient selection criteria, microbial identification methods, resistance testing protocols, and breakpoint standards across studies can significantly influence the microbial profiles detected. Furthermore, studies employ diverse microbial identification approaches, ranging from culture‐based methods to targeted molecular assays and next‐generation sequencing platforms, each with distinct sensitivities and limitations. Similarly, antimicrobial susceptibility testing methodologies and resistance gene detection strategies are not standardized, making direct comparisons between studies challenging and limiting translation into clinical guidelines. The establishment of harmonized standardized protocols for sample collection, microbial characterization, sequencing and resistance testing would improve reproducibility, facilitate cross‐study comparisons, and strengthen the evidence base required for clinical translation and guideline development.

Another important gap in the current evidence base is the predominance of cross‐sectional study designs, which provide only a snapshot of AMR patterns at a single time point. Longitudinal investigations are needed to better understand the temporal dynamics of resistance gene acquisition, persistence and dissemination within the periodontal microbiome. Such studies should evaluate changes in the resistome before and after periodontal interventions, including mechanical debridement and adjunctive antimicrobial therapy, while also considering patient‐specific factors such as age, systemic health, prior antibiotic exposure and geographic variation. Monitoring resistance profiles over time would provide critical insights into the ecological impact of periodontal treatment strategies and help determine the clinical significance of detected resistance genes. Ultimately, these data could inform precision prescribing practices and contribute to the development of more effective antimicrobial stewardship strategies in periodontology.

Routine assessment of the oral resistome before prescribing antibiotics may strengthen antimicrobial stewardship initiatives by enabling evidence‐based prescribing and reducing unnecessary antibiotic use. Incorporating microbiological diagnostics into clinical decision‐making could help identify resistance determinants in advance and guide the selection of more appropriate therapies. Moreover, recent advances in molecular diagnostics have expanded the ability to detect periodontal pathogens and associated AMR determinants with greater sensitivity and specificity than conventional culture‐based methods. Techniques such as PCR and shotgun metagenomic sequencing can identify resistance genes directly from clinical samples, providing valuable insights into the periodontal resistome. However, despite their diagnostic potential, these technologies remain largely confined to research settings because of their cost, technical complexity and limited accessibility in routine dental practice. Future developments should focus on the creation of rapid, affordable and user‐friendly chairside diagnostic platforms capable of detecting clinically relevant resistance markers in real time. Point‐of‐care (POC) molecular assays could support targeted antimicrobial prescribing, reduce unnecessary antibiotic exposure and enhance antimicrobial stewardship efforts within periodontal care, thereby facilitating a more personalized and evidence‐based treatment approach. The clinical relevance of these approaches is highlighted by studies investigating β‐lactamase‐producing anaerobic bacteria in chronic periodontitis. In one such study, 75% of patients characterized two species of β‐lactamase‐producing anaerobic bacteria within periodontal pockets, and molecular analysis demonstrated a high prevalence of the *cfxA* β‐lactamase gene among isolates, with *cfxA2* detected in 76.7% of strains, followed by *cfxA3* (14%) and *cfxA6* (9.3%) [99]. These findings suggest that resistance determinants are widespread among oral anaerobes and may contribute to antimicrobial treatment failure, underscoring the importance of accurate molecular detection methods.

Current recommendations suggest that POC resistance testing may be particularly valuable in complex clinical situations, including recurrent or persistent infections, cases where previous treatment has failed, immunocompromised patients, severe or rapidly progressing odontogenic infections and serious postoperative infections. Such applications may be especially relevant in specialist areas of dentistry, including oral and maxillofacial surgery and oral medicine, where severe infections often require more intensive management [100].

Although nucleic acid amplification‐based POC technologies are already used in medical settings for the diagnosis of infectious diseases and, in some cases, the detection of selected AMR genes, their implementation in dentistry remains limited [101]. This is partly because dental antibiotic prescribing relies on a relatively small range of antimicrobial agents, yet diagnostic platforms capable of rapidly identifying corresponding resistance markers are not widely available in routine practice. Existing dental POC tests primarily focus on host‐response protein biomarkers rather than microbial resistance profiles [102].

Advanced microfluidic systems integrating automated sample processing, DNA extraction, and real time PCR analysis have demonstrated the ability to detect oral pathogens directly from saliva samples. Because these platforms can be readily adapted by modifying target‐specific primers, they may provide a practical foundation for future chairside resistance screening [103]. However, successful integration into clinical practice will require appropriate implementation strategies, including practitioner education and training to ensure that microbiological findings are translated into informed treatment decisions [104].

Furthermore, within the framework of Oral One Health, resistance screening technologies could be integrated with advances in regenerative biology, biomaterials research, and digital dental technologies [105, 106, 107]. Such interdisciplinary approaches may enhance the management of oral diseases by combining molecular diagnostics with other innovative tools to support more precise, personalized and sustainable oral healthcare.

Advancing the field requires:

- Longitudinal follow‐up studies on periodontal patients that track the periodontal resistome before and after different types of periodontal therapy.
- Routine integration of open‐ended, high‐resolution tools such as shotgun metagenomics to map ARGs, MGEs and gene transfer networks.
- Standardization of sampling, sequencing, resistance testing and analytical methods.
- Resistome–phenotype correlation studies to determine when ARGs correspond to clinically relevant resistance.
- Consensus definitions of clinically meaningful resistance in the context of complex microbial communities.

These studies will have an impact on the current antimicrobial guidelines which may change our current concepts concerning the prevention, diagnosis and treatment of periodontal diseases. Comprehensive periodontal resistome mapping, across health, disease, treatment and diverse populations, will be central to shaping the next generation of periodontal diagnostics and therapeutics. Such work will refine our understanding of microbial ecology, allow stratification of patients by resistance risk, and inform precision prescribing strategies aligned with global antimicrobial stewardship priorities.

In summary, the future of periodontal therapy will involve the integration of population‐based evidence, high‐resolution microbial profiling and patient‐specific biological data. This hybrid model, supported by standardized methodology and robust resistome mapping, will help shift the field from empiric antibiotic use towards precision‐based, stewardship‐aligned periodontal care capable of mitigating AMR while improving clinical outcomes.

## Author Contributions

**Meera Alghafri:** writing – original draft, conceptualization, methodology, visualization. **Lindsey Edwards:** writing – review and editing, supervision, conceptualization, methodology. **David Moyes:** supervision, writing – review and editing. **Luigi Nibali:** supervision, writing – review and editing, funding acquisition, formal analysis.

## Funding

The authors have nothing to report.

## Conflicts of Interest

The authors declare no conflicts of interest.

## Data Availability

Data sharing not applicable to this article as no datasets were generated or analysed during the current study.
